# Neferine Protects against Hypoxic-Ischemic Brain Damage in Neonatal Rats by Suppressing NLRP3-Mediated Inflammasome Activation

**DOI:** 10.1155/2021/6654954

**Published:** 2021-05-08

**Authors:** Jin-jin Zhu, Bin-yuan Yu, Xiao-kai Huang, Min-zhi He, Bin-wen Chen, Ting-ting Chen, Huang-yi Fang, Shang-qin Chen, Xiao-qin Fu, Pei-jun Li, Zhen-lang Lin, Jiang-hu Zhu

**Affiliations:** ^1^Department of Neonatology, The Second Affiliated Hospital and Yuying Children's Hospital of Wenzhou Medical University, Wenzhou, Zhejiang 325027, China; ^2^Department of Hematology, The Second Affiliated Hospital and Yuying Children's Hospital of Wenzhou Medical University, Wenzhou, Zhejiang 325027, China

## Abstract

Hypoxic-ischemic encephalopathy (HIE) is recognized as the main cause of neonatal death, and efficient treatment strategies remain limited. Given the prevalence of HIE and the associated fatality, further studies on its pathogenesis are warranted. Oxidative stress and neuroinflammatory injury are two important factors leading to brain tissue injury and nerve cell loss in HIE. Neferine, an alkaloid extracted from lotus seed embryo, exerts considerable effects against several diseases such as cancers and myocardial injury. In this study, we demonstrated the neuroprotective effect of neferine on HIE and hypothesized that it involves the inhibition of neuronal pyroptosis, thereby ameliorating neurological inflammation and oxidative stress. We demonstrated that the mRNA levels of proteins associated with pyroptosis including caspase-1, the caspase adaptor ASC, gasdermin D, interleukin- (IL-) 18, IL-1*β*, and some inflammatory factors were significantly increased in neonatal HIBD model rats compared to those in the control group. The increase in these factors was significantly suppressed by treatment with neferine. We stimulated PC12 cells with CoCl_2_ to induce neuronal HIBD *in vitro* and investigated the relationship between neferine and pyroptosis by altering the expression of the NLRP3 inflammasome. The overexpression of NLRP3 partially reversed the neuroprotective effect of neferine on HIBD, whereas NLRP3 knockdown further inhibited caspase-1 activation and IL-1*β* and IL18 expression. In addition, simultaneous alteration of NLRP3 expression induced changes in intracellular oxidative stress levels after HIBD. These findings indicate that neferine ameliorates neuroinflammation and oxidative stress injury by inhibiting pyroptosis after HIBD. Our study provides valuable information for future studies on neferine with respect to neuroinflammation and pyroptosis.

## 1. Introduction

Hypoxic-ischemic encephalopathy (HIE) is the most common cause of death and disability among neonates, with a reported incidence of 2–3 cases per 1000 live births in developed countries and approximately 26 cases per 1000 live births in underdeveloped countries [1, 2]. In recent years, increased preemptive and neonatal care and improved critical care techniques have improved patient survival but they do not prevent neurological disorders, resulting in an increase in the incidence of such disorders in the adult population [3]. Surviving patients with HIE have lifelong neurological deficits, including cerebral palsy (10%–20%), auditory and visual problems (~40%), and motor and behavioral impairments, such as epilepsy, global developmental delay, and autism [2, 4, 5]. Therapeutic hypothermia has been established as a standard clinical treatment for newborns with moderate to severe HIE; however, it is only partially effective [6]. Therefore, more effective treatment strategies must be explored to improve the outcomes of HIE.

The pathogenesis of neonatal hypoxic-ischemic brain damage (HIBD) is complex and includes excitotoxicity, mitochondrial damage, inflammation, and oxidative stress, leading to progressive neuronal cell death in asphyxiated infants [2]. Fluctuations in blood flow in specific brain areas during the first few hours trigger excitotoxicity, free radical generation, and edema [7, 8]. The secondary phase of brain injury occurs within the next hours and days, leading to neuroinflammation, mitochondrial permeability loss, and cerebral autoregulatory function disturbance [9, 10]. A third stage has now been recognized, in which persistent inflammation may aggravate brain injury [11]. Therefore, alleviating cellular oxidative stress injury and inflammatory response and improving neuronal survival may serve as an effective therapeutic strategy in the treatment of neonatal HIBD.

Neuroinflammation plays an essential role in secondary neuronal injury and accounts for a considerable portion of the final brain cell loss in HIE [12]. Recently, pyroptosis has been identified as an inflammation-dependent type of programmed cell death. It is mediated by the inflammasome, which activates the caspase family proteins, resulting in the opening of cell membrane pores and cell swelling or even rupture, leading to the release of cellular contents including proinflammatory molecules [13]. The inflammasome consists of a pattern recognition receptor, the caspase adaptor ASC (also known as PYCARD), which contains a caspase activation and recruitment (CARD) domain, and caspase-1, which act as sensors of activated inflammasome [14]. Several inflammasomes have been identified; among which, the NLRP3 inflammasome in the nervous system has garnered considerable attention. Studies in neuronal models of cerebral ischemia *in vitro* and middle cerebral artery occlusion models in mice have indicated that ischemia leads to an increased expression of NLRP3 inflammasome components in the neurons and ischemic brain tissue and this phenomenon has been also observed in the brains of stroke patients [15, 16].

Neferine is a bisbenzylisoquinoline alkaloid isolated from lotus seed embryos that has various therapeutic properties including anticancer, antiarrhythmic, antithrombotic, and antithrombotic effects [17–20]. In addition, neferine protects against hypoxia-induced stress-mediated inflammation. In a Wistar rat liver ischemia model, it significantly inhibited the increase in aspartate aminotransferase, alanine aminotransferase, and hydroxyl radical levels, thereby exerting a protective effect [21]. In ischemic stroke, neferine has been found to exert a neuroprotective effect by modulating mitochondrial function [22]. In animal models of Alzheimer's disease, neferine reportedly exerted antiamnestic and antidepressive effects [23, 24]. Overall, these findings suggest that neferine may exert potent therapeutic effects. However, the role of neferine in the nervous system remains poorly understood and its potential molecular mechanism of action is unclear.

Although it has been found that in HIE, neural cells can induce neuroinflammatory responses, central inflammation is mainly mediated by microglia. However, whether it directly causes inflammatory damage to neural cells remains unknown and its underlying mechanism remains unexplored. Considering the role of pyroptosis in the inflammatory response, we hypothesized that NLRP3 inflammasome-mediated pyroptosis may be involved in the pathogenesis of neonatal HIBD. We used PC12, the rat pheochromocytoma cells, stimulated with CoCl_2_ to induce neuronal HIBD *in vitro* to explore whether neferine plays a protective role by inhibiting NLRP3-activated nerve cell pyroptosis. The PC12 cell line is commonly used in the study of neurons, and PC12 cells exposed to CoCl_2_ are a widely used model to study HIBD in neurological diseases. Further, we established a HIBD rat model to investigate whether neferine has a neuroprotective effect *in vivo* and whether pyroptosis is involved in nerve injury in HIE.

## 2. Materials and Methods

### 2.1. Neonatal HIBD Model Establishment

All animal care and experiments were performed in strict accordance with the Guidelines for the Care and Use of Laboratory Animals of the National Institute of Health and were approved by the Laboratory Animal Ethics Committee of Wenzhou Medical University (approval number: wydw2014-0058). Sprague–Dawley rats (200–250 g) were obtained from the Animal Center of the Chinese Academy of Sciences (Shanghai, China) and were maintained at the laboratory animal center at 23°C ± 2°C, 60% ± 10% humidity, and under a 12 h light/dark cycle. Adult rats had free access to standard food and drinking water and were allowed free mating to produce offspring for use in the experiments. The neonatal rat HIBD model was generated as described by Vannucci and Vannucci [25]. In total, 114 healthy pups were used in this study; each group comprised 38 pups. Postnatal (7-day-old) male rat pups were deeply anesthetized with 3% isoflurane, and the anesthesia was maintained with 1% isoflurane. The left common carotid artery was ligated; after which, the pups were placed with their mothers for 2 h. The pups were then maintained in a chamber filled with humidified gas (8% oxygen and 92% nitrogen; flow rate: 3 L/min) at 37.5°C for 2.5 h. In the sham-operated control group, the rats were not subjected to left carotid artery ligation or hypoxia.

### 2.2. Drug Administration Protocol

Neferine (MCE, USA) was dissolved in 10% dimethyl sulfoxide (DMSO) (Solarbio Biotechnology, Beijing, China) and 90% corn oil (Aladdin Industrial Corporation, Shanghai, China). The same solvent was used as the control. The drug delivery route and dose were selected based on a previous study [22]. Neferine-treated HIBD rats received 50 mg/kg neferine 1 h after HIBD by intragastrical gavage. Rats in the sham and HIBD groups received the same volume of solvent via intragastrical gavage.

### 2.3. Measurement of the Infarction Volume

The cerebral infarction volume was measured at 24 h after HIBD induction by 2,3,5-triphenyltetrazolium chloride (TTC, Sigma-Aldrich, St. Louis, MO, USA) staining of the brain. The pups in each group were deeply anesthetized and perfused with 10 mL of 0.9% normal saline. The brains were harvested, frozen at –80°C for 6 min, and cut into coronal sections of 2 mm. The sections were incubated in 1% TTC dissolved with PBS in the dark at 37°C for 20 min and then immersed in 4% paraformaldehyde (PFA) overnight. The infarction volume was measured using ImageJ software (National Institutes of Health, Bethesda, MD, USA).

### 2.4. Measurement of Brain Water Content

The brain water content was measured using the dry-wet ratio method as previously described [26]. The brain tissues were obtained at 24 h after HIBD, and the left (i.e., injured) hemisphere was immediately isolated and weighed to obtain the wet weight. The tissues were then dried in an electrothermal oven at 100°C for 72 h to measure the dry weight. The brain water content was calculated as follows: (wet weight–dry weight)/wet weight × 100%.

### 2.5. Real-Time Reverse Transcription Polymerase Chain Reaction (RT-qPCR)

The total RNA was extracted from brain tissues using TRIzol reagent (Roche, South San Francisco, CA, USA). The RNA concentration was determined based on the optical density (OD) 260/OD280 ratio; the OD was determined using an ultraviolet spectrophotometer. The RNA was reverse transcribed into cDNA, and qPCR was performed using the CFX96 Optics Module (Bio-Rad, Singapore). The thermal cycling conditions were as follows: 95°C for 3 min; 95°C for 10 s and 60°C for 30 s (40 cycles); and 95°C for 1 min and decreased to 65°C at a gradient of 0.5°C (41 cycles; melting curve step). Primers (Table 1) were designed based on cDNA sequences obtained from GenBank. Target gene expression was quantified using the 2^–ΔΔCT^ method, with *β*-actin mRNA as the internal control.

### 2.6. Assessment of Oxidative Stress

Brain samples were collected at 24 h after HIBD and homogenized, and then, the protein concentrations were determined. Superoxide dismutase (SOD) and glutathione peroxidase (GSH-Px) activities were measured by using commercial kits (Solarbio Biotechnology, Beijing, China) per the manufacturer's instructions.

### 2.7. Histological Staining and Immunohistochemistry

Seven days after HIBD, three rats in each group were perfused with 20 mL of saline and 15 mL of 4% PFA. The brains were immersed in 4% PFA at 4°C for 24 h, embedded in paraffin, and sectioned. Parts of the sections were stained with Nissl stain (Solarbio, Beijing, China) according to the manufacturer's instructions and captured using a digital scanner (HS6; Leica, Solms, Germany). The others were used for immunohistochemical staining. The sections were boiled in citrate buffer for 10 min for antigen retrieval and then incubated with 10% BSA at 37°C for 1 h. The sections were incubated with anti-MAP-2 (1 : 200, Sc-20172; Santa Cruz Biotechnology) and anti-MBP (1 : 200, Sc-13914; Santa Cruz Biotechnology) at 4°C overnight. The sections were then incubated with horseradish peroxidase-conjugated donkey goat secondary antibody (1 : 1000, Sc-2020; Santa Cruz Biotechnology) at 37°C for 1 h. Finally, the sections were incubated with 3,3′-diaminobenzidine. Images were acquired using a digital scanner (Leica HS6, Germany).

### 2.8. Cell Culture and Treatment

Differentiated PC12 cells were purchased from Type Culture Collection of Chinese Academy of Sciences (Shanghai, China). The cells were cultured in DMEM supplemented with 10% FBS (both from Gibco, Grand Island, NY, USA), in a humidified incubator (5% CO_2_, 37°C).

Neferine was dissolved in DMSO at a stock concentration of 50 mM. Based on the results of preliminary testing of a concentration gradient, cells were treated with the optimal concentration of 10 nM neferine in the subsequent experiments. *In vitro* cytotoxicity studies were performed after hypoxia induction of cells using CoCl_2_. PC12 cells were treated with 1 mM CoCl_2_ or with 1 mM CoCl_2_ + 10 nM neferine for 24 h. Control cells (not stimulated with CoCl_2_ and neferine) were incubated with medium containing DMSO (0.1% *v*/*v*).

### 2.9. Terminal Deoxynucleotidyl Transferase dUTP Nick-End Labeling (TUNEL) Staining

Apoptotic DNA fragmentation was analyzed using an *in situ* cell death detection kit (Roche, USA). For *in vivo* analysis, the brains were collected at 24 h after HIBD, embedded in paraffin, sectioned, and treated with 20 *μ*g/mL proteinase K (ST533, Beyotime, Beijing, China) at 37°C for 15–30 min. *In vitro*, PC12 cells were collected 24 h after CoCl_2_ insult, fixed with 4% PFA for 1 h, and then incubated with 0.1% Triton X-100 in 0.1% sodium citrate at 4°C for 3 min. The cells and tissue sections were incubated with the TUNEL reaction mixture in the dark in a humidified chamber at 37°C for 1 h. The sections were then washed with PBS and stained with DAPI. The cells were stained green, whereas the nuclei were stained blue. The cells and sections were analyzed and imaged using a fluorescence microscope (Ni-U; Nikon, Tokyo, Japan). The apoptotic index was determined as follows: apoptotic index = number of apoptotic cells/total number of cells × 100.

### 2.10. Immunofluorescence Staining

For the *in vivo* analysis, the brains were collected from the rats 24 h after HIBD, embedded in paraffin, sectioned, and dehydrated for antigen retrieval. For the *in vitro* experiments, PC12 cells were collected 24 h after CoCl2 injury and fixed in 4% PFA for 1 h. Both cells and tissue sections were incubated with 5% goat serum for 1 h at room temperature. The sections were incubated with NLRP3 (1 : 200, DF7438; Affinity Biosciences, Cincinnati, OH, USA), caspase-1/P20/P10 (1 : 200, 22915-1AP; ProteinTech) at 4°C overnight. After being incubated with the appropriate secondary antibody (1 : 200, Cell Signaling Technology) at 37°C for 1 h, the sections were washed with PBS and stained with DAPI. The cells and sections were analyzed and imaged using a fluorescence microscope (Ni-U; Nikon).

### 2.11. Short Interfering (Si) RNA and Plasmid Transfection

PC12 cells were transfected with 10 mM NLRP3-targeting siRNA (5′-GCAATGCCCTTGGAGACAT-3′; RiboBio, Guangzhou, China) or normal control siRNA using Lipofectamine 3000 reagent (Invitrogen, Carlsbad, CA, USA). NLRP3 cDNA was cloned into the pcDNA 3.1 plasmid vector. At 48 h after transfection, the cells were incubated in the indicated medium with 1 mM CoCl_2_ for 24 h.

### 2.12. Cell Viability Assay

The Cell Counting Kit-8 (CCK8) (C0038, Beyotime, China) was used to measure cell viability. Cells were plated in 96-well plates at a density of 1 × 10^5^ cells/well and cultured for 24 h. The cells were treated with CoCl_2_ or CoCl_2_ + neferine for another 24 h. Thereafter, 10% volume of CCK-8 solution was added to each well and mixed well; the plate was incubated at 37°C in the dark for 30 min. The OD450 was measured using a microplate reader, and the results are expressed as the percentage of viable cells relative to the number of cells observed in the control group.

### 2.13. Measurement of Intracellular Reactive Oxygen Species (ROS) Production

A ROS assay kit (DCFH-DA; Beyotime, Shanghai, China) was used to assess intracellular ROS levels. After treatments, adherent PC12 cells were washed with DMEM and stained with 5 *μ*M DCFH-DA at 37°C for 30 min and then washed three times with PBS. The labeled PC12 cells were trypsinized, washed twice with PBS, and analyzed using FlowJo 7.6.1 software.

### 2.14. Measurement of Mitochondrial ROS

Mitochondrial oxidative stress levels were analyzed using MitoSOX Red (Yeasen, Shanghai, China), a fluorescent mitochondrial superoxide anion indicator. In brief, the treated PC12 cells were washed with Hank's balanced salt solution (HBSS) without Ca^2+^ or Mg^2+^ and incubated with 5 *μ*M MitoSOX Red at 37°C in the dark for 30 min. The cells were washed with HBSS, and the nuclei were stained with DAPI. The cells were then observed under a fluorescence microscope (Ni-U).

### 2.15. Western Blotting

Rats under deep anesthesia were decapitated at 24 h and 7 days after HIBD. Brain tissues were collected and extracted in radioimmunoprecipitation (RIPA) (P0013B; Beyotime) buffer with a phenylmethylsulfonyl fluoride (PMSF) protease inhibitor, and the sample was centrifuged at 13201 × *g*, for 30 min at 4°C. The cells were washed extensively with PBS and incubated with RIPA buffer containing PMSF and protease inhibitors on ice for 30 min. The proteins were quantified using bicinchoninic acid reagent (P0012S; Beyotime), and equal amounts of protein (45 mg) were separated by 10% sodium dodecyl sulfate-polyacrylamide gel electrophoresis and transferred onto polyvinylidene difluoride membranes (Bio-Rad, Hercules, CA, USA) with a pore size of 0.45 mm. After blocking with 5% skimmed milk for 2 h, the membranes were incubated with primary antibodies at 4°C overnight. We used primary antibodies against B-cell lymphoma-2 (Bcl-2; 1 : 1000, #2876; Cell Signaling Technology, Danvers, MA, USA), Bax (1 : 500, 50599-2-Ig; ProteinTech, Rosemont, USA), MAP-2 (1 : 1000, #4542S; Cell Signaling Technology), MBP (1 : 1000, ab40390; Abcam, Cambridge, UK), NLRP3 (1 : 1000, ab263899; Abcam), caspase-1/P20/P10 (1 : 1000, 22915-1AP; ProteinTech), ASC/TMS1 (1 : 1000, A16672; ABclonal, Wuhan, China), GSDMD (1 : 1000, ab219800; Abcam), IL-1*β* (1 : 1000, AF5103; Affinity Biosciences, Cincinnati, OH, USA), IL-18 (1 : 1000, 10663-1-AP; ProteinTech), and *β*-actin (1 : 5000, 66009–1 Ig; ProteinTech). The membranes were washed three times in Tris-buffered saline with Tween 20 and then incubated with goat anti-rabbit IgG (1 : 4000, #7074; Cell Signaling Technology) or goat anti-mouse IgG (1 : 4000, #7076; Cell Signaling Technology) at room temperature for 2 h. Signals were detected using the ChemiDoc XRS+ Imaging System (Bio-Rad). All experiments were repeated at least thrice, and the protein bands were analyzed by densitometry using Image Lab 5.0 software (Bio-Rad).

### 2.16. Statistical Analysis

The experiments were performed in triplicates and repeated at least three times. The data are presented as mean ± standard deviation (SD). The data were analyzed using a one-way analysis of variance (ANOVA) followed by Fisher's least significant difference or Bonferroni's post hoc test (equal variance) or Dunnett's T3 post hoc test (unequal variance) for multiple comparisons. Student's *t*-test was used to compare two groups. Statistical analyses were conducted using version SPSS 19.0 (SPSS, Chicago, IL) or GraphPad 8.0 (GraphPad Software, San Diego, CA, USA). Results with *P* < 0.05 were considered statistically significant.

## 3. Results

### 3.1. Neferine Attenuates Acute HIBD in Neonatal Rats

**Figure 1:**
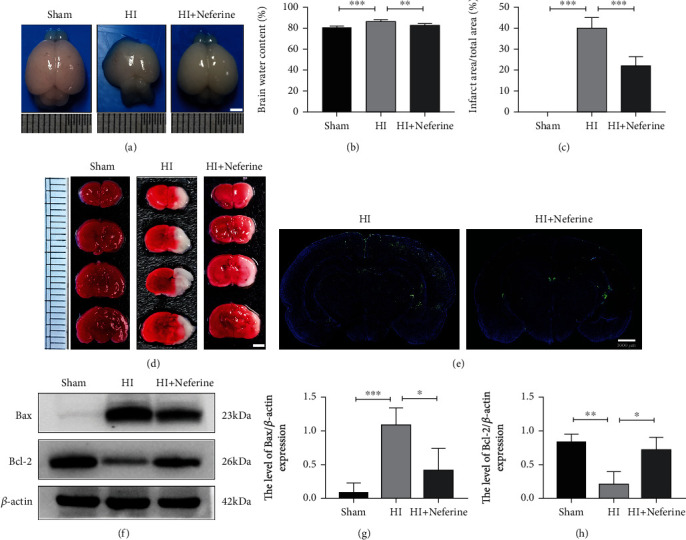
Neferine attenuates acute HI brain damage in neonatal rats. (a) The brains were isolated from each group 24 h after HI brain injury. (b) The ratio of wet and dry is calculated in each group. ^∗∗^*P* < 0.01 and ^∗∗∗^*P* < 0.001. *n* = 5. (c) Calculation of the infarct area shown by TTC staining. ^∗∗∗^*P* < 0.001. *n* = 4. (d) Representative results of TTC-stained coronal brain sections 24 h after HI brain injury. (e) Representative images of TUNEL staining (green). The nucleus (blue) was labeled with DAPI 24 h after HI brain injury. Scale bar = 1000 *μ*m. *n* = 3. (f) Protein expression level of Bax and Bcl-2 24 h after HI brain injury. (g, h) Analyses of Bax and Bcl-2 (of *β*-actin). ^∗^*P* < 0.05, ^∗∗^*P* < 0.01, and ^∗∗∗^*P* < 0.001. *n* = 3.

The water content was measured in the brains collected from rats in the sham, HIBD, and HIBD + neferine groups (Figures 1(a) and 1(b)). The results showed that neferine intervention significantly reduced brain edema caused by HIBD. Next, we quantitatively measured the cerebral infarction volume in the three groups (Figures 1(c) and 1(d)). TTC staining showed that neferine significantly reduced the cerebral infarct area after HIBD. We used the TUNEL assay and Western blotting to investigate whether neferine could reduce neuronal apoptosis after HIBD. In the TUNEL-stained whole-brain scan images, the number of TUNEL-positive cells was significantly higher in the HI-injured (left) hemibrain than in the noninjured (right) hemibrain, whereas this increase was significantly suppressed by neferine intervention (Figure 1(e)). Accordingly, the Western blotting analysis revealed that neferine significantly increased the expression of the antiapoptotic protein Bcl-2 after HIBD, whereas it inhibited the expression of the proapoptotic protein Bax (Figures 1(f)–1(h)). Together, these findings indicated that neferine attenuated nerve injury in the acute phase of HIBD.

### 3.2. Neferine May Reduce Neuron Loss and Promote White Matter Recovery

**Figure 2:**
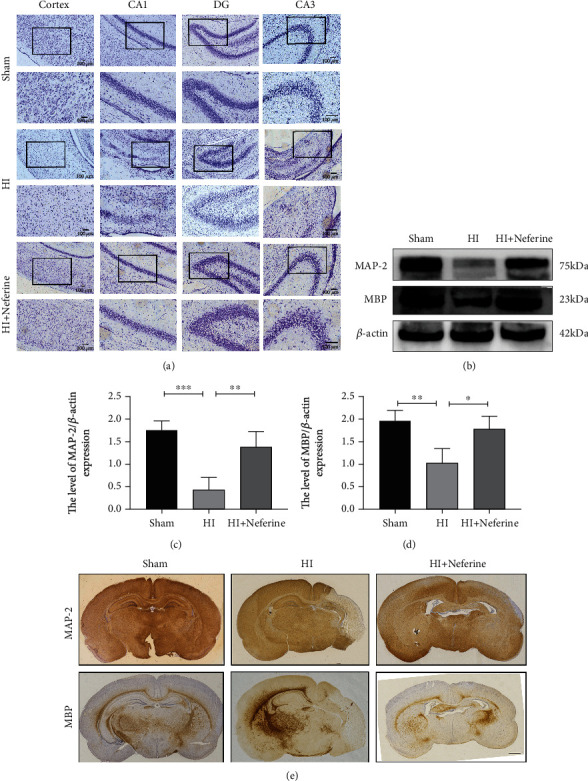
Neferine may reduce neuron loss and promote white matter recovery. (a) Representative images of Nissl staining in the cortex and hippocampus CA1, CA3, and DG in the injured ipsilateral brain 7 d post-HI. The columns on the right show magnified images of the black boxes in the left column. Scale bar = 100 *μ*m. (b) Protein expression level of microtubule-associated protein 2 (MAP-2) and myelin basic protein (MBP) 24 h after HI brain injury 7 d post-HI. (c, d) Analyses of MAP-2 and MBP (of *β*-actin). ^∗^*P* < 0.05, ^∗∗^*P* < 0.01, and ^∗∗∗^*P* < 0.001. *n* = 4. (e) Representative images of immunohistochemical staining of MAP-2 and MBP 7 d post-HI. Scale bar = 1 mm.

We performed Nissl staining at 7 days after HIBD to observe neuronal arrangement and Nissl body integrity in brain tissues (Figure 2(a)). In the sham group, neurons in the cortex and the CA1, CA3, and DG regions of the hippocampus were regularly arranged and shaped and numerous large Nissl bodies were observed around the nucleus. However, rats in the HIBD group exhibited disorganized neurons with pyknotic nuclei, as well as a considerable loss of neurons in some locations. Neferine treatment suppressed these phenomena and significantly increased the number of neurons and Nissl bodies.

We used immunohistochemical staining and Western blotting to detect the expression of MAP-2, a neuronal marker, and MBP, an oligodendrocyte marker, to investigate whether neferine could accelerate remyelination and axonal repair in neonatal HI-injured rats (Figures 2(b)–2(e)). The expression of MAP-2 and MBP in the HIBD group was significantly lower than that in the sham group, whereas neferine significantly increased the expression of both proteins. Taken together, these results suggest that neferine reduced neuronal loss, promoted morphological recovery of the brain tissue, promoted myelination, and reduced white matter injury in rat brain tissue.

### 3.3. Neferine May Reduce Acute Inflammation and Oxidative Stress after HIBD in Neonatal Rats

**Figure 3:**
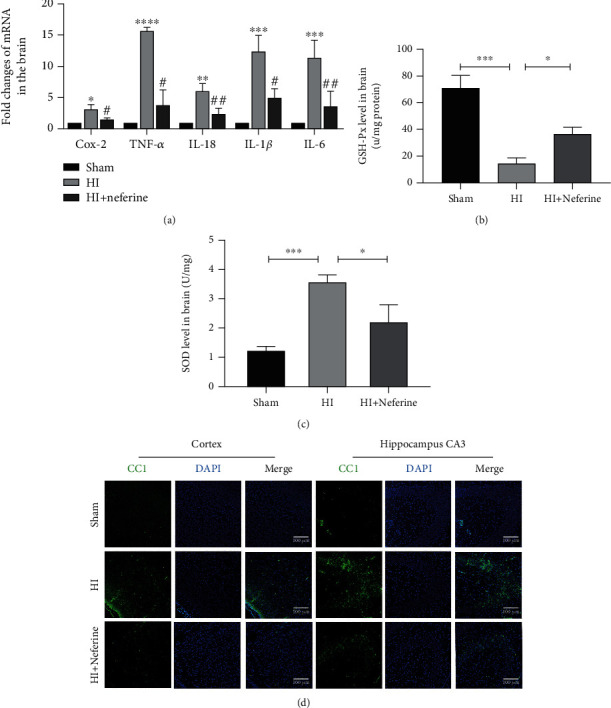
Neferine may reduce acute inflammation and oxidative stress after HIBD in neonatal rats. (a) The levels of mRNA expression in brain tissue 24 h after HI brain injury normalized to those of *β*-actin for each sample. ^∗^*P* < 0.05, ^∗∗^*P* < 0.01, ^∗∗∗^*P* < 0.001, and ^∗∗∗∗^*P* < 0.0001 versus the sham group. ^#^*P* < 0.05 and ^##^*P* < 0.01 versus the HI group. *n* = 3. (b) The SOD activity in the brain tissue 24 h after HI brain injury. ^∗^*P* < 0.05 and ^∗∗∗^*P* < 0.001. *n* = 3. (c) The GSH-px level in brain tissue 24 h after HI brain injury. ^∗^*P* < 0.05 and ^∗∗∗^*P* < 0.001. *n* = 3. (d) Representative immunofluorescence staining images of cleaved caspase-1 (green) and DAPI (blue) 24 h after HI. Scale bar = 100 *μ*m.

The cellular antioxidant defense system protects cells from ROS produced during metabolism, and it has been reported that neferine has strong antioxidant properties [27, 28]. Therefore, we investigated whether neferine has a neuroprotective role in HIBD by reducing inflammation and reducing oxidative stress. We examined the mRNA expression of inflammatory markers after HIBD by RT-qPCR (Figure 3(a)). The mRNA levels of *Cox-2*, *TNF-α*, *IL-18*, *IL-1β*, and *IL-6* in the HIBD group were significantly higher than those in the sham group. However, the mRNA levels of these inflammatory factors were significantly reduced by neferine.

SOD is a vital antioxidant enzyme that protects cells from hypoxic injury by scavenging superoxide anion radicals [29]. GSH-px is the major nonenzymatic antioxidant that protects cells from oxidative damage by preventing intracellular oxygen radical accumulation [30]. The SOD and GSH-px levels indicated that neferine attenuated oxidative stress damage in the brain tissue after HIBD (Figures 3(b) and 3(c)).

### 3.4. Neferine Attenuates NLRP3/Caspase-1/IL-1*β* Activation in Neonatal Rat Brain Tissue after HIBD

**Figure 4:**
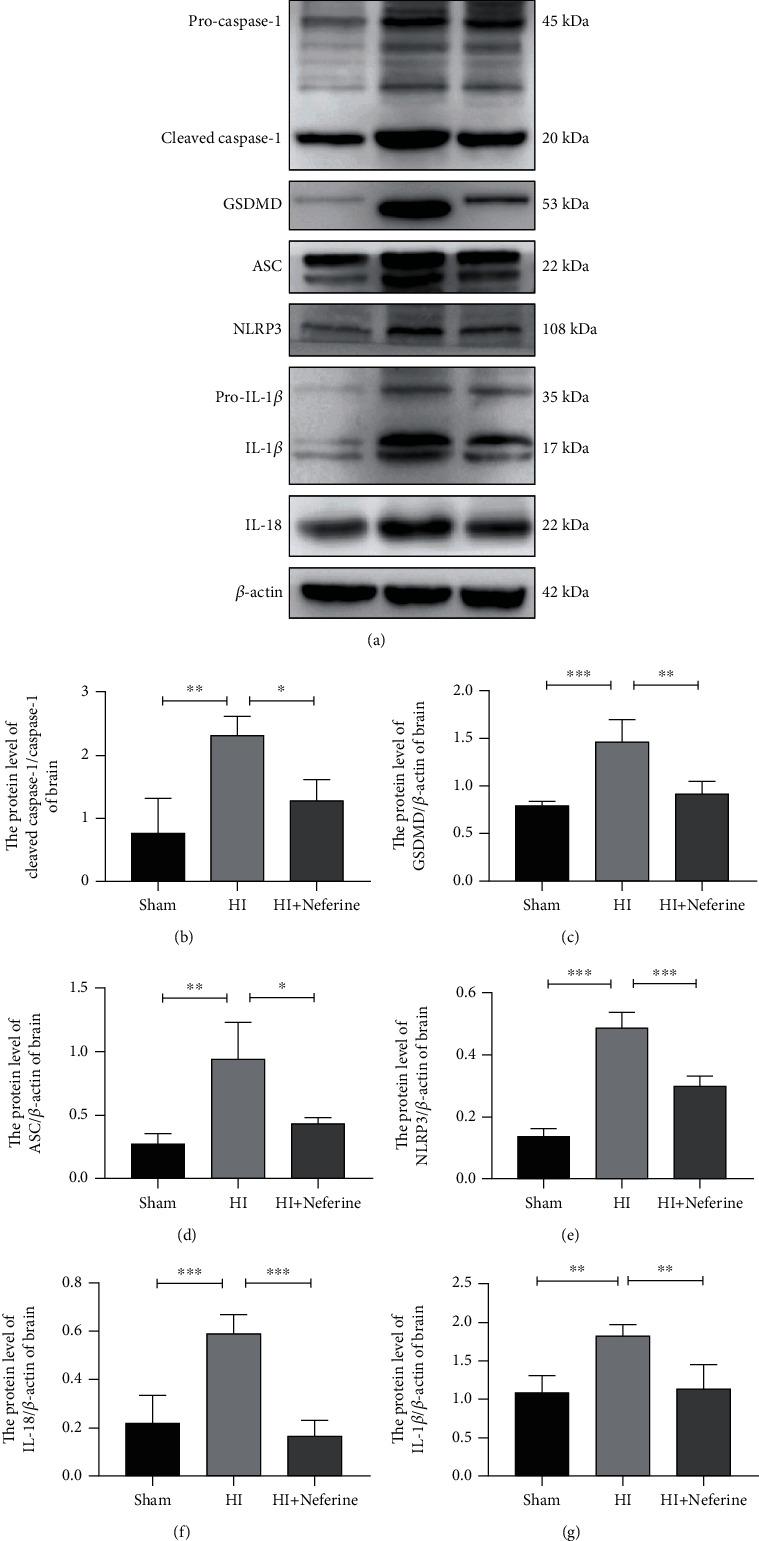
Neferine attenuates NLRP3/caspase-1/IL-1*β* activation in neonatal rat brain tissue after HI. (a) The protein levels of cleaved caspase-1, GSDMD, ASC, NLRP3, IL-1*β*, and IL-18 were evaluated by Western blotting in brain tissues. (b–g) Analyses of cleaved caspase-1, GSDMD, ASC, NLRP3, IL-1*β*, and IL-18 (normalized to *β*-actin). ^∗^*P* < 0.05, ^∗∗^*P* < 0.01, and ^∗∗∗^*P* < 0.001. *n* = 4.

NLRP3 inflammatory bodies are multiprotein complexes composed of NLRP3, ASC, and caspase-1. The NLRP3 inflammasome plays a key role in the development of inflammatory responses in the central nervous system [31], and its hyperactivation mediates the activation of downstream signaling pathways involved in various neurological diseases [32]. Therefore, we investigated the effect of neferine on the NLRP3/caspase-1/IL-1*β* signaling pathway by Western blotting and RT-qPCR (Figures 4(a)–4(g)). As expected, neferine treatment inhibited the activation of NLRP3 and caspase-1 in the brain tissues of HIBD rats (Figures 4(b) and 4(e)). In addition, neferine treatment substantially suppressed the mRNA and protein expression of IL-18 and IL-1*β* in the brain tissues of HIBD rats (Figures 3(a), 4(f), and 4(g)). The secretion of IL-18 and IL-1*β* requires GSDMD, which upon inflammasome activation, is translocated to the plasma membrane, where it forms pores through which these cytokines are released [33]. Accordingly, we found increased GSDMD expression after HIBD (Figure 4(c)). Furthermore, we found that cleaved caspase-1 expression significantly increased in the cortex and hippocampus after HIBD in rats, whereas neferine could suppress this phenomenon (Figure 3(d)).

### 3.5. Neferine Effectively Reduces CoCl_2_-Induced PC12 Cell Injury

**Figure 5:**
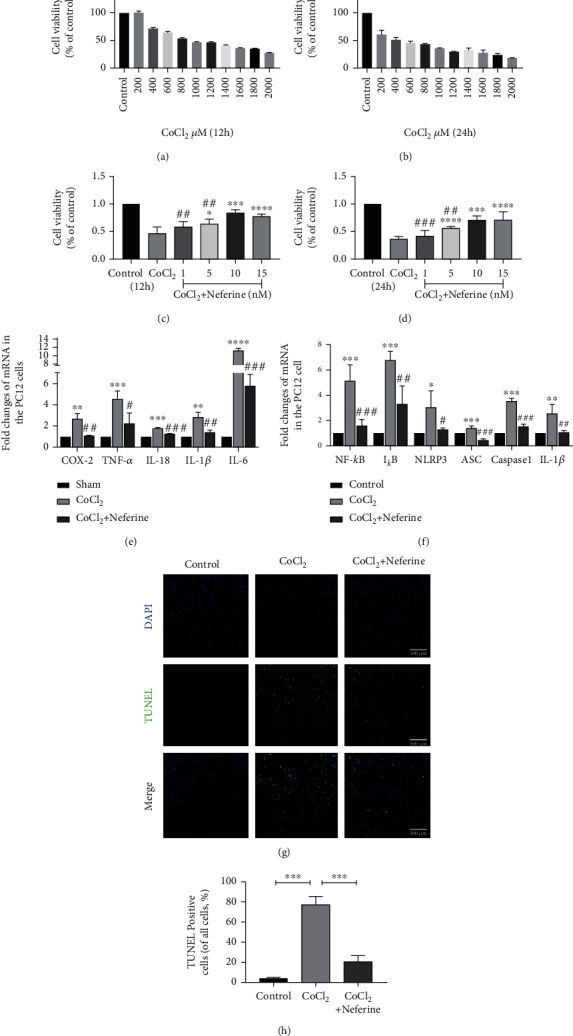
Neferine effectively reduces CoCl_2_-incuded PC12 cell injury. (a, b) PC12 cells were treated for 12 h and 24 h in a CoCl_2_ dose-dependent manner as assessed with CCK8. (c, d) PC12 cells were treated with different concentrations of neferine for 12 h and 24 h simultaneously under 1000 m*Μ* CoCl_2_ treatment, and cell viability was determined by CCK8. (e, f) The levels of each mRNAs in PC12 were normalized to those of *β*-actin for each group. ^∗∗^*P* < 0.01, ^∗∗∗^*P* < 0.001, and ^∗∗∗∗^*P* < 0.0001 versus the sham group. ^#^*P* < 0.05, ^##^*P* < 0.01, and ^###^*P* < 0.001 versus the HI group. *n* = 3. (g) Representative images of TUNEL staining (green). The nucleus (blue) was labeled with DAPI in PC12. Scale bar = 100 *μ*m. (h) Quantification of TUNEL staining in PC12. ^∗∗∗^*P* < 0.001. *n* = 4.

The effect of CoCl_2_ on PC12 cell viability was examined using the CCK8 assay. CoCl_2_ (0.1–1 nM; 24 h) significantly decreased cell viability in a time- and dose-dependent manner compared with the control (Figures 5(a) and 5(b)). The optimal dose of neferine to effectively protect PC12 cells from 1 mM CoCl_2_-induced damage was found to be 10 nm (Figures 5(c) and 5(d)). The RT-qPCR analysis of multiclock inflammatory factors revealed that neferine pretreatment decreased inflammatory factor production and protected against CoCl_2_-induced PC12 cell injury (Figures 5(e) and 5(f)). TUNEL staining indicated that neferine reduced CoCl_2_-stimulated apoptosis (Figures 5(g) and 5(h)).

### 3.6. Changes in NLRP3 Inflammasome Expression Alter the Effects of Neferine on CoCl_2_-Induced Pyroptosis and Oxidative Stress

**Figure 6:**
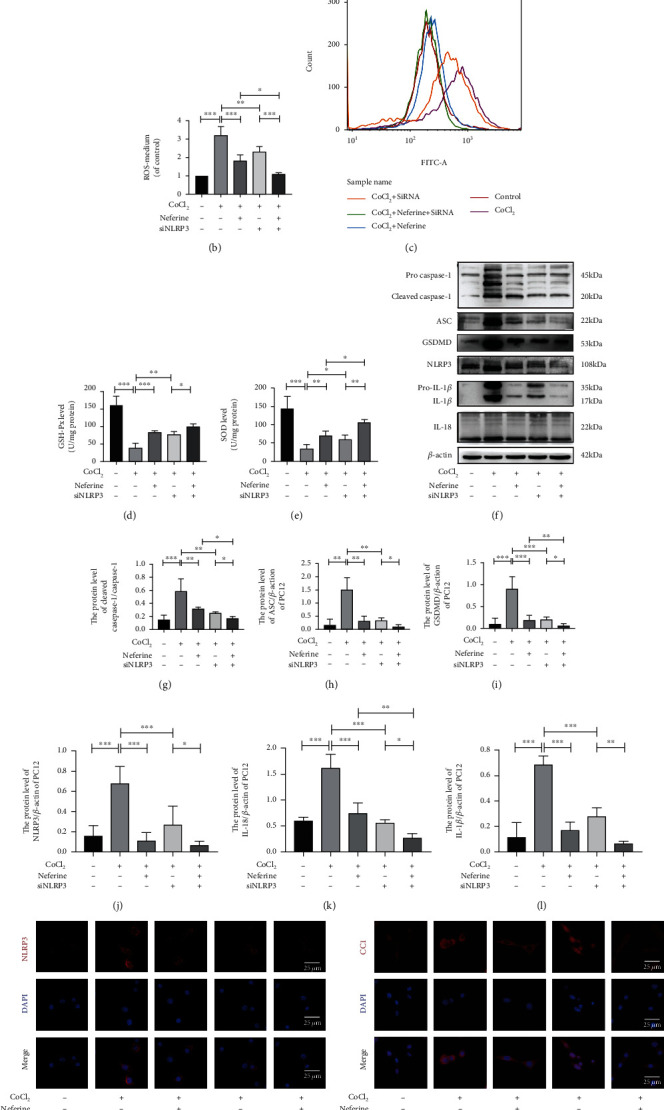
Silencing NLRP3 could enhance the inhibitory effect of neferine on CoCl_2_-induced PC12 pyroptosis and oxidative stress. (a) Mitochondrial ROS generation of each group was detected by MitoSox (red) and DAPI (blue) staining. (b, c) Intracellular ROS was analyzed using flow cytometry after DCF-DA staining. ^∗^*P* < 0.05, ^∗∗^*P* < 0.01, and ^∗∗∗^*P* < 0.001. *n* = 3. (d) The GSH-px level in PC12. ^∗^*P* < 0.05, ^∗∗^*P* < 0.01, and ^∗∗∗^*P* < 0.001. *n* = 4. (e) The SOD level in PC12. ^∗∗^*P* < 0.01 and ^∗∗∗^*P* < 0.001. *n* = 4. (f) The protein levels of cleaved caspase-1, GSDMD, ASC, NLRP3, IL-1*β*, and IL-18 were evaluated by Western blotting in PC12. (g–l) Analyses of cleaved caspase-1, GSDMD, ASC, NLRP3, IL-1*β*, and IL-18 (of *β*-actin). ^∗^*P* < 0.05, ^∗∗^*P* < 0.01, and ^∗∗∗^*P* < 0.001. *n* = 4. (m, n) Representative immunofluorescence staining images of NLRP3 and cleaved caspase-1 (red) in PC12. The nucleus (blue) was labeled with DAPI. Scale bar = 20 *μ*m.

**Figure 7:**
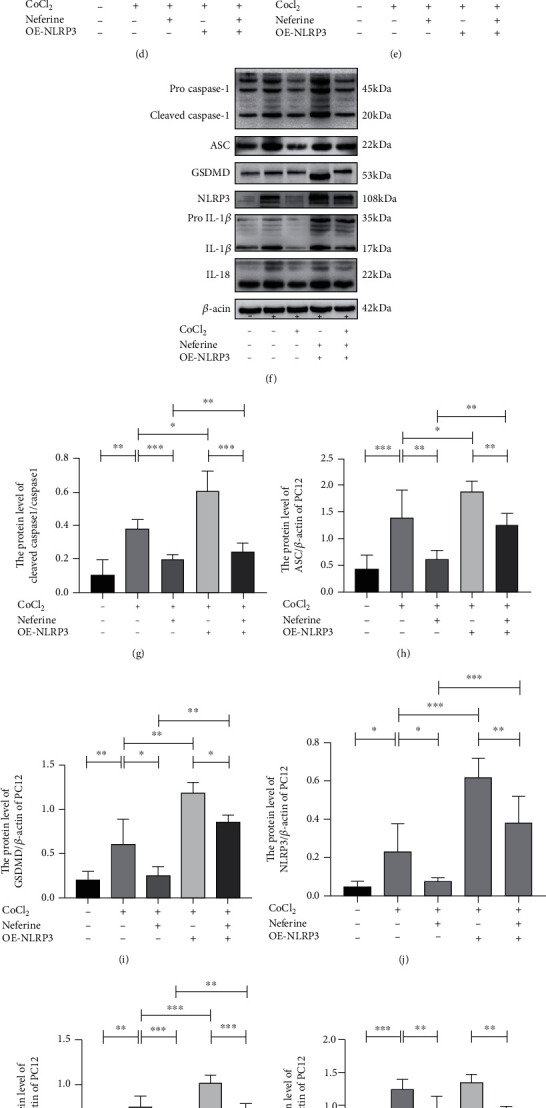
Overexpress NLRP3 partially reversed the protection of neferine against Cocl_2_-induced PC12 pyroptosis and oxidative stress. (a) Mitochondrial ROS generation of each group was detected by MitoSox (red) and DAPI (blue) staining. (b, c) Intracellular ROS was analyzed using flow cytometry after DCF-DA staining. ^∗∗^*P* < 0.01 and ^∗∗∗^*P* < 0.001. *n* = 3. (d) The GSH-px level in PC12. ^∗^*P* < 0.05, ^∗∗^*P* < 0.01, and ^∗∗∗^*P* < 0.001. *n* = 4. (e) The SOD level in PC12. ^∗^*P* < 0.05. *n* = 4. (f) The protein levels of cleaved caspase-1, GSDMD, ASC, NLRP3, IL-1*β*, and IL-18 were evaluated by Western blotting in PC12. (g–l) Analyses of cleaved caspase-1, GSDMD, ASC, NLRP3, IL-1*β*, and IL-18 (of *β*-actin). ^∗^*P* < 0.05, ^∗∗^*P* < 0.01, and ^∗∗∗^*P* < 0.001. *n* = 4. (m, n) Representative immunofluorescence staining images of NLRP3 and cleaved caspase-1 (red) in PC12. The nucleus (blue) was labeled with DAPI. Scale bar = 25 *μ*m.

On the basis of the above finding, that is, neferine acted as a strong antioxidant, we hypothesized that neferine may reduce cellular HIBD by inhibiting mitochondrial oxidative stress and ROS production, thereby suppressing NLRP3 inflammasome activation. Therefore, we explored the role of NLRP3 in neferine function by silencing or overexpressing NLRP3. We used the MitoSOX fluorescent probe assay to detect superoxides in the mitochondria and DCFH-DA fluorescence to determine the level of total intracellular ROS in each group (Figures 6(a)–6(c) and 7(a)–7(c)). We found that neferine pretreatment decreased the level of oxidative stress induced by CoCl_2_ in PC12 cells. In addition, pretreatment with neferine significantly increased CoCl_2_-suppressed SOD and GSH-px production and NLRP3 silencing significantly enhanced this response, whereas the overexpression of NLRP3 had the opposite effect (Figures 6(d), 6(e), 7(d), and 7(e)). NLRP3 silencing significantly inhibited CoCl_2_-induced pyroptosis and the expression of injury-related proteins, including NLRP3, cleaved caspase-1, ASC, GSDMD, IL-1*β*, and IL-18, whereas the overexpression of NLRP3 had the opposite effect (Figures 6(f)–6(l) and 7(f)–7(l)). Similar results were obtained for NLRP3 and caspase-1 expression by immunofluorescence (Figures 6(m), 6(n), 7(m), and 7(n)). These results indicated that neferine reduces CoCl2-induced cell pyroptosis and inflammatory factor release by suppressing the NLRP3 inflammasome.

## 4. Discussion

**Figure 8:**
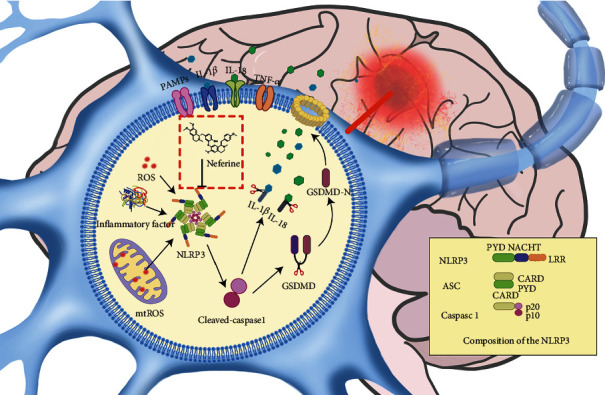
Mechanism diagram of neferine in HIE. Neferine inhibits NLRP3 inflammasome-activated cell injury by attenuating cellular oxidative stress damage after HI.

HIE is one of the major causes of neonatal neurological disease and death worldwide, but currently, there is no effective treatment. Substantial preclinical evidence supports the view that a neuroinflammatory response is the main pathophysiological factor causing perinatal cerebral HIBD [34–36]. Several studies have revealed that NLRP3-activated inflammasome in the nervous system activates caspase-1 to cleave IL-18, which triggers neural cells to undergo pyroptosis dependent on inflammatory responses, causing nerve injury [37, 38]. In this study, we found that NLRP3-activated inflammasome is involved in nerve injury after HIBD, which paves the way to a new, promising potential therapeutic treatment of HIE in the future (Figure 8).

Neferine, a natural compound extracted from green seed embryos of lotus plants, has been confirmed to be nontoxic to various rat organs, including the liver, kidney, and brain [22, 39]. There are numerous studies on neferine in the context of myocardial ischemia and cancer research; however, its role and action mechanism in brain injury remain unclear. It has been suggested that neferine exerts neuroprotection, improves cognition, and acts as an antidepressant [24, 40]. Here, we investigated the potential protective effect of neferine on HIBD using *in vitro* and *in vivo* models and explored the possible underlying mechanisms. Neferine treatment reduced brain edema and the cerebral infarction volume and inhibited neuroinflammation and oxidative stress injury in rats in a short period. In addition, we found that neferine improved the structure of brain tissues and the recovery of white matter in the long term. In *in vitro* CoCl_2_-stimulated PC12 cells, neferine promoted cell survival. Compared with neferine alone, CoCl_2_-induced cell death increased with simultaneous neferine treatment and NLRP3 expression plasmid transfection, whereas the opposite phenomenon occurred upon simultaneous administration of neferine and NLRP3 siRNA. Finally, we demonstrated that neferine exerts its neuroprotective effect by inhibiting neuronal NLRP3 inflammasome activation. Thus, our results showed that neferine may be beneficial for the recovery of neonatal HIBD.

The brain of newborns is particularly vulnerable to oxidative stress because unsaturated fatty acids and metal ions that catalyze oxygen radical reactions are abundant, whereas the antioxidant levels are low [41]. Oxidative stress and ROS produced under HIBD cause severe oxidative damage to lipids, proteins, and nucleic acids. Accordingly, we found that the activities of SOD and GSH-px were significantly decreased after HIBD *in vitro* and *in vivo* and that the total cellular ROS levels increased *in vitro*; neferine treatment could significantly suppress this response. Mitochondria perform several important functions, and numerous studies have suggested that mitochondrial dysfunction results in the production of large amounts of ROS after HIBD, which cannot be immediately removed; they accumulate excessively due to metabolic interruption, thereby playing a central role in HIBD-induced neurodegeneration [42]. Using a MitoSOX fluorescent probe to detect mitochondrial ROS *in vitro*, we found that red fluorescence was significantly higher in CoCl_2_-stimulated PC12 cells, which was reversed by neferine treatment. Together, these results support our hypothesis that neferine exerts a neuroprotective effect by suppressing oxidative stress damage after HIBD.

We then explored the potential mechanisms of oxidative stress-induced nerve injury and death. Several studies have revealed that intracellular ROS production and intracellular Ca^2+^ mobilization promote the production of NLRP3 inflammatory bodies [43]. We hypothesized that neferine exerts its neuroprotective effect by inhibiting the activation of NLRP3 inflammatory bodies after HIBD. Yuan et al. found that NLRP3, caspase-1, GSDMD, and IL-1B were increased to varying levels in peripheral blood samples from patients with different degrees of HIE, and that NLRP3 started to increase at 12 h after HIBD in a rat model [44]. In line with this, we observed an increase in NLRP3 inflammatory bodies and pyrogen-related gene and protein expression at 24 h after HIBD induction. Interestingly, by immunofluorescence staining, we found that NEU-N-expressing cells also expressed NLRP3, which we suspect is age related. The neonatal brain is more sensitive to the internal environment of the nervous system, and while the specific mechanism requires further research, recent studies suggest that neurons may express NLRP3 [45, 46].

The activation of NLRP3 inflammasome may also be an initiation step, induced by the congenital immune sensor NOD-like receptors. In the NLRP3 inflammasome, NLRP3 acts as the receptor, ASC/PYCARD as the linker, and caspase-1 as the effector [47]. The ASC domain mediates the activation of inactive proproteinase 1, asparagin-1, which completes the NLRP3 inflammasome complex. Activated inflammatory caspase-1 cleaves GSDMD, and the gasdermin-N domain formed acts on the cell membrane to form pores, driving pyroptosis and triggering inflammation [48]. Cleaved caspase-1 in turn activates IL-18 and IL-1*β*. Both L-18 and IL-1*β* promote the accumulation of ROS, which in turn stimulates the activation of asparagin-1 and the NLRP3 inflammasome, resulting in the production of more IL-18, activation of pyroptosis, and establishment of a feedback loop between NLRP3 inflammasome activation and oxidative stress [49]. Excessive and sustained NLRP3 inflammasome stimulation may lead to cell death by activating pyrophosphate mechanisms. In the central nervous system, the IL-18 and IL-1*β* receptors are expressed on the nerve cell surface, and an increase in these proinflammatory cytokines has been observed in cerebrospinal fluids of infants with HIE [50]. IL-1*β* may induce neuronal injury through various mechanisms, including the induction of nitric oxide production, activation of necrotic and apoptotic pathways, modulation of synaptic plasticity, and effects on components of the mitogen-activated protein kinase pathway, which controls neurodegeneration [51]. Here, we found that treatment with neferine significantly reduced NLRP3 inflammasome activation and inhibited ASC, GSDMD, and cleaved caspase-1 activation, thereby reducing IL-1*β* and IL18 expression in the HIBD rat model. This corroborated that neferine may exert its neuroprotective effect by inhibiting the stimulation of the NLRP3 inflammasome.

Considering the effect of neferine on NLRP3 inflammasome activation, we further investigated the involvement of NLRP3 inflammasome activation in the *in vitro* CoCl_2_-stimulated PC12 cell model by altering NLRP3 expression. Interestingly, the neuroprotective effect of neferine was abrogated upon NLRP3 overexpression and the knockdown of NLRP3 inflammasome expression further inhibited caspase-1 activation and IL-1*β* and IL18 expression. In addition, simultaneous alteration of NLRP3 expression caused changes in intracellular oxidative stress levels after HIBD. These results suggest that the inhibition of NLRP3 activation may be critical for the neuroprotective effect of neferine after HIE.

This study had some limitations. We did not investigate whether neferine provides long-term neuroprotection after HIBD, including motor, learning, and other abilities. We hypothesize that by reducing NLRP3 activation in the neonatal brain, the inhibition of IL-1*β* expression would substantially ameliorate epilepsy after HIBD, as well as improve learning and memory abilities. Furthermore, we believe that the effect of neferine on HIE may be multifaceted, warranting more studies.

## 5. Conclusions

This study provides comprehensive evidence supporting the potential beneficial effects of neferine on HIE and the potential protective mechanisms of this alkaloid. Our data suggest that neferine inhibits NLRP3 inflammasome-activated cell injury by attenuating cellular oxidative stress damage after HIBD. Apparently, the effect of neferine is not limited to a single pathway but the inhibition of pyroptosis is likely to be a major contributor to its therapeutic effect. While future clinical applications of neferine require further research, it may serve as a promising neuroprotective agent for HIE.

## Figures and Tables

**Table 1:** Primers used in the polymerase chain reaction.

Gene	Forward primers	Reverse primers
*IL-6*	GAGTTGTGCAATGGCAATTC	ACTCCAGAAGACCAGAGCAG
*TNF-α*	TACTCCCAGGTTCTCTTCAAGG	GGAAGGCTGACTTTCTCCTGGTA
*COX2*	CGGAGGAGAAGTGGGGTTTAGGAT	TGGGAGGACTTGCGTTGATGG
*IL-18*	AAACCCGCCTGTGTTCGA	GGGTTCCATGGTGAAGTCAAC
*IL-1β*	CACCTCTCAAGCAGAGCCACAG	GGGTTCCATGGTGAAGTCAAC

IL: interleukin; TNF-*α*: tumor necrosis factor-alpha.

## Data Availability

The data used to support the findings of this study are available from the corresponding author with reasonable request.
